# AP-1^cFos/JunB^/miR-200a regulate the pro-regenerative glial cell response during axolotl spinal cord regeneration

**DOI:** 10.1038/s42003-019-0335-4

**Published:** 2019-03-06

**Authors:** Keith Z. Sabin, Peng Jiang, Micah D. Gearhart, Ron Stewart, Karen Echeverri

**Affiliations:** 1 Department of Genetics, Cell Biology and Development, University of Minnesota, Minneapolis, MN 55455 USA; 2 Morgridge Institute for Research, Madison, 53715 WI USA; 3 Present Address: Marine Biological Laboratory, Eugene Bell Center for Regenerative Biology and Tissue Engineering, Woods Hole, 02543 MA USA

## Abstract

Salamanders have the remarkable ability to functionally regenerate after spinal cord transection. In response to injury, GFAP^+^ glial cells in the axolotl spinal cord proliferate and migrate to replace the missing neural tube and create a permissive environment for axon regeneration. Molecular pathways that regulate the pro-regenerative axolotl glial cell response are poorly understood. Here we show axolotl glial cells up-regulate AP-1^cFos/JunB^ after injury, which promotes a pro-regenerative glial cell response. Injury induced upregulation of miR-200a in glial cells supresses *c-Jun* expression in these cells. Inhibition of miR-200a during regeneration causes defects in axonal regrowth and transcriptomic analysis revealed that miR-200a inhibition leads to differential regulation of genes involved with reactive gliosis, the glial scar, extracellular matrix remodeling and axon guidance. This work identifies a unique role for miR-200a in inhibiting reactive gliosis in axolotl glial cells during spinal cord regeneration.

## Abstract (web summary)

Keith Sabin et al. showed that upregulation of the AP-1 complex, composed of c-Fos and JunB, in the axolotl spinal cord promotes a pro-regenerative glial cell response. This response is impaired by inhibition of miR-200a; suggesting an important role for this microRNA in axolotl spinal cord regeneration.

## Introduction

Salamanders have retained the remarkable ability to functionally regenerate after spinal cord injury (SCI)^1–9^. In response to SCI, glial fibrillary acidic protein (GFAP)^+^ glial cells proliferate and migrate through the lesion to create a permissive environment for axon regeneration^9–12^. This is in stark contrast to the mammalian response to SCI where damaged astrocytes undergo reactive gliosis and contribute to the glial scar by secreting axon growth inhibitory proteins like chondroitin sulfate proteoglycans (CSPGs) and collagens^13–16^. The glial scar is a complex subject, it has been shown to be beneficial by preventing more damage to the spinal cord but it also expresses proteins that are inhibitory to axon regeneration^16^. Many different vertebrate animals, in addition to salamanders; have the ability to regenerate a functional spinal cord after injury, including lamprey, xenopus and zebrafish. Common to all these animals is that regeneration occurs in the absence of reactive gliosis and glial scar formation^10–12,17^. The molecular pathways that promote functional spinal cord regeneration without glial scar formation are poorly understood.

Recent advances in molecular genetics and transcriptional profiling techniques are beginning to elucidate the molecular and cellular responses necessary for functional spinal cord regeneration. Lampreys, which represent the most basal vertebrate ancestor that diverged from a shared common ancestor to humans more than 560 million years ago, can regenerate locomotive function within 12 weeks of a full spinal cord transection. After SCI in lamprey resident GFAP^+^ astrocytes elongate and form a glial bridge that facilitates axons to regenerate through the lesion^18–26^. This is reminiscent of the injury-induced glial bridge formed by GFAP^+^ glial cells in zebrafish spinal cord, which is similarly necessary for axon regeneration^27,28^. Xenopus display robust functional spinal cord regeneration in the larval stages by activating the GFAP^+^/Sox2^+^ glial cells to divide, migrate, and repair the lesion which allows axons to regenerate. However the tadpoles ability to regenerate is lost after metamorphoses into an adult frog^29–41^. Similar events occur in axolotl, GFAP + /Sox2 + cells adjacent to the injury site are activated in response to injury and will migrate to repair the lesion, however axolotls can regenerate throughout life^4,7–10,42^. In axolotls an injury to the spinal cord is fully repaired, rostral and caudal sides of the spinal cord reconnect but there is no glial bridge structure formed as is seen in zebrafish^43^. A common theme in these species is the absence of reactive gliosis and the lack of a glial scar. To facilitate functional recovery these remarkable animals activate glial cells to regenerate the ependymal tube or form a glial bridge both of which act as a highway to guide axon regeneration through the lesion site.

In contrast mammalian glial cells; often referred to as astrocytes; undergo a process of reactive gliosis in response to injury. Historically, reactive astrocytes were characterized as highly proliferative, hypertrophic cells that express high levels of GFAP. Advances in lineage tracing and transcriptomic profiling approaches have revealed a much higher degree of heterogeneity among reactive astrocytes^44,45^. Recent publications suggest that reactive astrocytes and components of glial scar are beneficial for mitigating the inflammatory response, resulting in less neuronal death early after injury^46–48^. However, the chronic persistence of the glial scar remains a major barrier to axon regeneration. Despite the high degree of heterogeneity across reactive astrocytes, several injury models have identified a critical role for the transcriptional complex AP-1 in promoting reactive gliosis by activating the GFAP promoter and other downstream pathways leading to glial scar formation^49–54^.

AP-1 is commonly formed as a heterodimeric complex of FOS and JUN proteins capable of regulating the expression of various genes involved with cell cycle, extracellular matrix remodeling and cell migration^55–58^. Research from several labs has shown that while Jun family members can homodimerize; c-Fos is an obligate heterodimer^59–62^. The identity of AP-1 target genes and the ability of AP-1 to transcriptionally activate or repress target genes is partially dependent on the combination of FOS and JUN proteins that comprise the AP-1 dimer^57,63–65^. Interestingly, after CNS injury in mammals both c-Fos and c-Jun are upregulated in reactive astrocytes and function to promote reactive gliosis and glial scar formation^49–51,66^. After SCI, axolotl glial cells upregulate expression of c-Fos, Fos protein is found in cells adjacent to the injury site for 1 day post injury. Inhibition of c-Fos expression leads to defects in spinal cord regeneration^11^. Additionally, inhibition of Jun N-terminal kinase (JNK) blocks spinal cord regeneration in axolotl^11^. Unlike mammals, these results highlight an important, pro-regenerative role for AP-1 during axolotl spinal cord regeneration. However in axolotl the identity of the c-Fos binding partner was unknown.

Here we identify that axolotl glial cells express a non-canonical AP-1^cFos/JunB^ after SCI. Axolotl glial cells up-regulate miR-200a expression, which directly represses c-Jun expression during spinal cord regeneration, thereby blocking the formation of AP-1^cFos/cJun^. Furthermore, chronic overexpression of AP-1^cFos/cJun^ in axolotl glial cells leads to defects in axon regeneration. Specific inhibition of miR-200a in axolotl glial cells partially phenocopied AP-1^cFos/cJun^ overexpression leading to defects in axon regeneration. Transcriptomic profiling of control or miR-200a inhibitor electroporated spinal cords identified differential expression of genes indicative of reactive gliosis and glial scar formation. Our data support a role for the non-canonical AP-1^cFos/JunB^ in promoting the pro-regenerative glial cell response necessary for spinal cord regeneration in axolotl.

## Results

### Glial cells up-regulate the non-canonical AP-1^cFos/JunB^

**Fig. 1:**
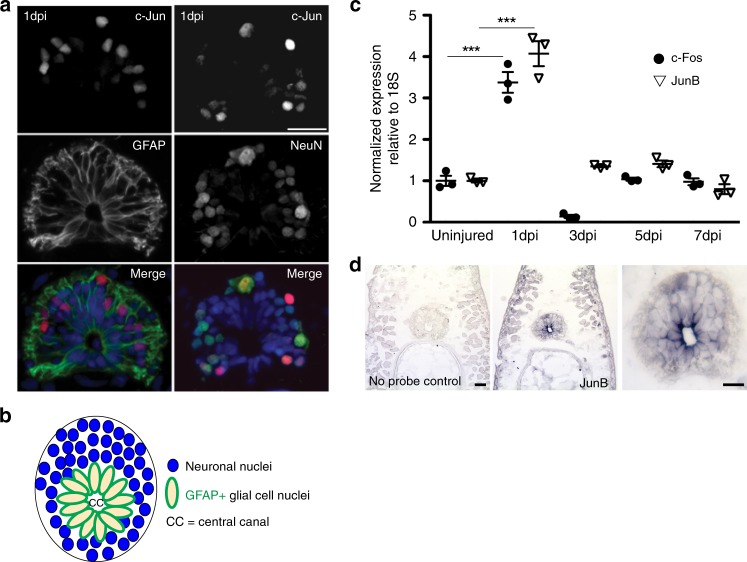
Axolotl glial cells express AP-1^cFos/JunB^ after spinal cord injury. **a** Immunohistochemical analysis of regenerating spinal cords at 1 day post injury shows only NeuN^+^ neurons express c-Jun. GFAP^+^ glial cells are negative for c-Jun expression (*n* = 5) Scale bars = 50 μm. **b** Schematic diagram of the structure of the axolotl spinal cord, neuronal cell bodies that surround the glial cells are shown in blue, glial cells line the central canal (CC), they have a large nucleus and express GFAP on the membrane (green). **c** qRT-PCR profiling shows upregulation of c-Fos and JunB during axolotl spinal cord regeneration (*n* = 3). **d** In situ hybridization confirms JunB expression in glial cells at 1 day post injury, higher magnification image of panel **d**, showing JunB transcript in the oval-shaped glial cells that line the central canal (*n* = 5) Scale bars = 50 μm. ***p ≤ 0.001. Error bars represent ± S.T.D

We have previously shown that the transcription factor c-Fos is upregulated in GFAP^+^ glial cells in axolotl 1 day post SCI and is necessary for glial cell division after injury^11^. However, c-Fos functions as an obligate heterodimer but the identity of the c-Fos-binding partner in axolotl glial cells was unknown^11^. To determine if the canonical AP-1^cFos/cJun^ is expressed in axolotl glial cells, we performed immunofluorescent staining for c-Jun at 1 day post injury in axolotl spinal cords. This analysis revealed that c-Jun is not upregulated in GFAP^+^ glial cells after injury but is strongly expressed in NeuN^+^ neurons (Fig. 1a). This suggests that axolotl glial cells do not up-regulate the canonical AP-1^cFos/cJun^ after injury but instead must up-regulate a different JUN family member.

To discover putative c-Fos-binding partners, we mined previously published transcriptional profiling data of axolotl spinal cord regeneration^11^. This approach identified JunB as a potential c-Fos-binding partner due to its similar transcriptional dynamics after SCI. Subsequent qRT-PCR confirmed that *JunB* is upregulated after SCI, which mirrors the expression dynamics of *c-Fos* (Fig. 1c). In situ hybridization confirmed that *JunB* is expressed in glial cells, which line the central canal at 1 day post injury, these cells are easily identifiable due to their oval shape and position (Fig. 1b schematic, 1d JunB staining). Finally, to confirm that axolotl c-Fos and JunB interact biochemically we utilized the biotin based BioID system^67^. Axolotl JunB-BioID showed robust c-Fos pull down, confirming their specific biochemical interaction (Supplementary Figure 1A). Taken together, these results support our hypothesis that after SCI, axolotl glial cells primarily form the non-canonical AP-1^cFos/JunB^ heterodimer.

### Differential activity of AP-1 complexes at the GFAP promoter

**Fig. 2:**
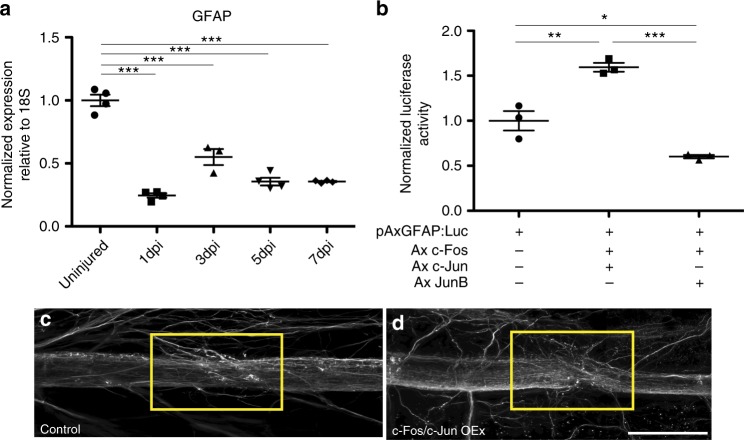
AP-1^cFos/JunB^ differentially regulates GFAP expression. **a** qRT-PCR data showing down regulation of *GFAP* throughout the time course of spinal cord regeneration (*n* = 4). **b** Luciferase reporter experiments carried out in a neural progenitor cell line, B35 cells, show different activation levels of the axolotl GFAP promoter depending on the AP-1 complex present. Axolotl AP-1^cFos/JunB^ represses the axolotl GFAP promoter, while AP-1^cFos/cJun^ activates the GFAP promoter (*n* = 3). **c**, **d** Whole mount antibody staining of β-III tubulin, overexpression of axolotl AP-1^cFos/cJun^ inhibits axon regeneration at 7 days post injury (**d**) compared to control injured animals (**c**). Yellow box indicates the area of injury (control *n* = 11, AP-1^cFos/cJun^ OE *n* = 13). *p ≤ 0.05, **p ≤ 0.01, ***p ≤ 0.001. Error bars represent ± S.T.D. Scale bar = 75 μm

After SCI in mammals, damaged mammalian astrocytes become reactive, upregulate *GFAP* expression and contribute to glial scar formation^15,16^. Previous work in axolotl suggests that after spinal cord injury GFAP^+^ glial cells in fact downregulate many intermediate filament proteins including GFAP^68^. We used qRT-PCR to assay *GFAP* expression during axolotl spinal cord regeneration and confirmed that in our transection model GFAP levels are downregulated after injury (Fig. 2a). These results suggest that axolotl glial cells undergo a fundamentally different molecular response to injury than mammalian astrocytes. Interestingly, AP-1^cFos/cJun^ directly regulates *GFAP* expression during reactive gliosis in mammals^51^. Therefore, we wanted to test if a different composition of the AP-1 complex can lead to differential GFAP promoter activity.

We cloned a fragment of axolotl GFAP promoter, that is the equivalent region of the human promoter which is sufficient to drive GFP expression specifically in axolotl glial cells^11,69^. We cloned this axolotl GFAP promoter upstream of a luciferase reporter and co-transfected neural progenitor cells with axolotl AP-1^cFos/cJun^ or axolotl AP-1^cFos/JunB^. As expected, the canonical AP-1^cFos/cJun^ is a strong transcriptional activator of the GFAP promoter (Fig. 2b). Interestingly as we hypothesized AP-1^cFos/JunB^, actively repressed the axolotl GFAP promoter in the luciferase assay (Fig. 2b). Taken together, these results suggest that non-canonical AP-1^cFos/JunB^ may function to mitigate reactive gliosis and glial scar formation in axolotl glial.

Finally, we wanted to determine if chronic over expression of AP-1^cFos/cJun^ in axolotl glial cells is sufficient to affect axolotl spinal cord regeneration. Electroporation was used to introduce GFP-tagged c-Fos and c-Jun overexpression constructs into the glial cells of the spinal cord^70^. After 24 h, animals were screened for GFP^+^ glial cells and animals with > 70% labeled glial cells were selected for SCI. The animals were harvested 7 days post injury, a time point at which axons have regenerated though the lesion^10,11^, and the extent of axon regeneration was examined by whole mount immunofluoresence for β-III tubulin. In control spinal cords, the β-III tubulin^+^ axons had regenerated through the lesion as indicated by the yellow rectangle (Fig. 2c). Conversely, when AP-1^cFos/cJun^ was overexpressed in the glial cells, this lead to defective axon regeneration, some axons sprouted randomly and did not grow directly through the lesion site, as indicated by the yellow box (Fig. 2d). This phenotype could be indicative of an overall growth repulsive environment^10^ that may be reminiscent of the mammalian glial scar. The phenotype observed is not a complete inhibition of axonal regeneration and could be due to a competition between the endogenous JunB and the overexpressed c-Jun for binding to the endogenous c-Fos. This would result in some glial cells being led to a pro-regenerative fate while others are directed towards a less regenerative direction due to the presence of the c-Fos:c-Jun heterodimer.

### miR-200a represses c-Jun expression in axolotl glial cells

Our previous work showed that c-Jun is highly upregulated in NeuN^+^ neurons and in dorsal root ganglia close to the injury site but is not upregulated after injury in glial cells (Fig. 1a)^42^. Work from our lab and many others have shown that dynamic regulation of target gene expression by microRNAs (miRNA) is critical for functional tissue regeneration^10,71–76^. Therefore, we wanted to test the hypothesis that axolotl glial cells repress c-Jun expression via a miRNA mediated mechanism. Analysis of the c-Jun 3’ untranslated region (UTR) using TargetScan identified a conserved miR-200 seed sequence across many species, including axolotl. While miR-200 family members are known to play a pivotal role during neurodevelopment^77^, little is known about their function during regeneration.

**Fig. 3:**
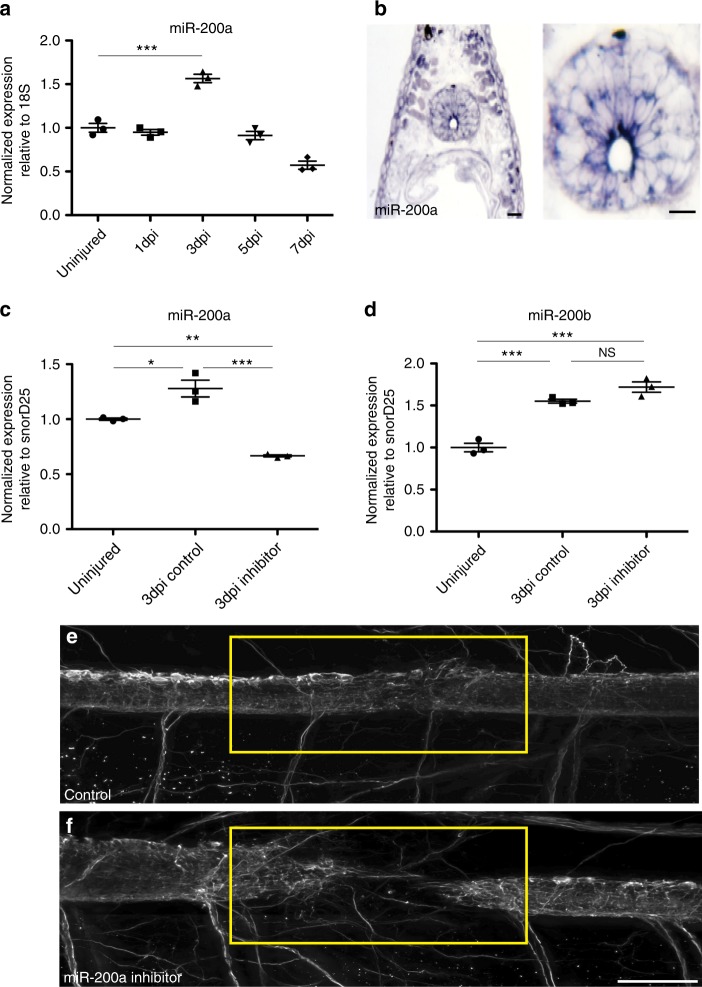
Upregulation of miR-200a in glial cells after spinal cord injury is required for spinal cord regeneration. **a** qRT-PCR analysis shows that miR-200a is upregulated at 3 days post injury (*n* = 3). **b** In situ hybridization confirms miR-200a expression specifically in glial cells at 3 days post injury in the regenerating spinal cord. The adjacent panel is a higher magnification of just the spinal cord from panel **b**, showing miR-200a expression in the oval shaped glial cells that line the central canal (*n* = 6). Scale Bar = 50 μm. **c** qRT-PCR analysis confirms knockdown of miR-200a after spinal cords were injected with a miR-200a inhibitor (*n* = 3). **d** miR-200a inhibition does not affect miR-200b levels (*n* = 3). **e** Whole mount β-III tubulin staining at 7 days post injury in control animals shows regeneration of axons through the lesion. **f** Inhibition of miR-200a results in defects in axon regeneration through the injury site in comparison to control animals. Yellow box indicates the injury site, (controls *n* = 33, miR-200a inhibitor *n* = 35). *p ≤ 0.05, **p ≤ 0.01, ***p ≤ 0.001, N.S. is not significant. Error bars represent ± S.T.D. Scale Bar = 75 μm

**Fig. 4:**
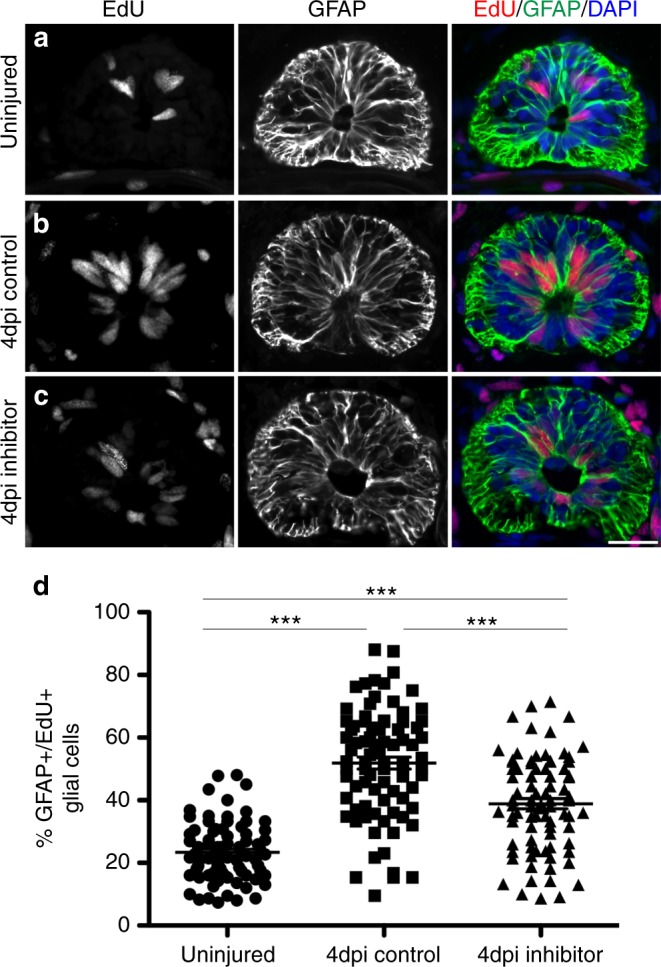
Inhibition of miR-200a leads to decreased glial cell proliferation after injury. EdU incorporation experiments revealed a homeostatic level of proliferative GFAP^+^ glial cells in the spinal cord. **a** Homeostatic levels of cell proliferation in uninjured spinal cord; **b** proliferation of GFAP^+^ cells increases 4 days post injury. **c** Inhibition of miR-200a results in a significant decrease in the percent of proliferative GFAP^+^ glial cells at 4 days post injury. Scale bar = 50 μm. **d** Quantification of the percent of EdU^+^/GFAP^+^ glial cells in uninjured, 4dpi control and 4dpi inhibitor spinal cords (*n* = 8). ***p ≤ 0.001. Error bars represent ± S.T.D. Scale Bar = 75 μm

A time course analysis revealed that miR-200a is expressed at homeostatic levels in the uninjured spinal cord and is significantly upregulated at 3 days post injury (Fig. 3a). In situ hybridization confirmed that miR-200a is specifically expressed in glial cells during axolotl spinal cord regeneration (Fig. 3b), panel 3b shows a higher magnification image of the spinal cord, miR-200a is expressed in the oval glial cells lining the central canal. To determine whether miR-200a function is necessary for functional spinal cord regeneration we injected and electroporated spinal cords with a commercially available chemically synthesized miR-200a inhibitor that knocked down miR-200a levels but did not affect a closely related family member, miR-200b (Fig. 3c, d). Whole mount β-III tubulin staining revealed miR-200a inhibition resulted in a complete failure of axons to regenerate through the lesion by 7 days post injury compared to controls (Fig. 3e, f). Furthermore, axons were observed sprouting from the spinal cord both rostral and caudal to the lesion, similar to regeneration defects observed with AP-1^cFos/cJun^ over expression (Fig. 2d). Histological analysis of the control versus inhibitor treated animals also revealed a failure of the rostral and caudal sides of the spinal cord to reconnect (Supplementary Figure 2). This failure of reconnection, which is necessary to facilitate growth of axons through the lesion site may be due to perturbation in proliferation of the GFAP^+^ glial cells after injury. Proliferation of the glial cells 500 µm rostral and caudal to the injury site was quantified by counting the number of GFAP^+^/EdU^+^ cells at 4 days post injury. This analysis revealed that even in the uninjured spinal cord there is a small population of proliferative glial cells (Fig. 4a, d) which significantly increased by 4 days post injury in control spinal cords (Fig. 4b, d) Inhibition of miR-200a led to a significant decrease in proliferation of glial cells after injury (Fig. 4c, d). In axolotl it is known that reconnection of the rostral and caudal sides of the neural tube is necessary for the axons to regrow through the injury site^2,8,9,42^.

**Fig. 5:**
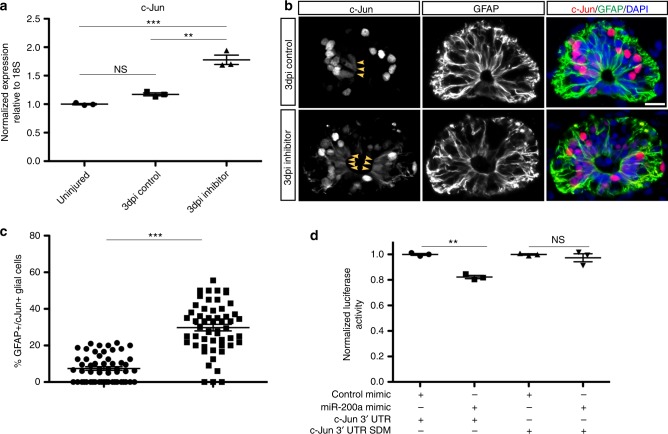
miR-200a directly represses c-Jun expression in GFAP^+^ glial cells during spinal cord regeneration in vivo. **a** qRT-PCR analysis of c-Jun levels after spinal cord injury shows that miR-200a inhibition leads to a significant increase in *c-Jun* expression (*n* = 3). **b** Antibody staining of GFAP and c-Jun in axolotl spinal cord sections. Inhibition of miR-200a leads to increased expression of c-Jun in GFAP^+^ glial cells, panel **b** bottom row (*n* = 8). Scale Bar = 50 μm. **c** Quantification of the percent of c-Jun^+^/GFAP^+^ glial cells after inhibition of miR-200a. **d** miR-200a functionally targets the 3’UTR of c-Jun, co-transfection of a miR-200a mimic and a luciferase reporter containing the axolotl c-Jun 3’ UTR confirms that miR-200a actively represses c-Jun. Mutation of the miR-200a seed sequence in the axolotl c-Jun 3’ UTR alleviates that repression (**d**), (*n* = 3). **p ≤ 0.01, ***p ≤ 0.001. Error bars represent ± S.T.D. In **b** arrowheads indicate the Jun^+^/GFAP^+^ glial cells, N.S. not significant

To determine whether c-Jun expression is affected by miR-200a inhibition we assayed for changes in c-Jun transcript abundance with qRT-PCR at 3 days post injury, the time point miR-200a is most highly expressed (Fig. 3a), and found an increase in c-Jun expression compared to control and uninjured spinal cords (Fig. 5a). In addition inhibition of miR-200a in an uninjured spinal cord also causes a significant increase in c-Jun expression **(**Supplementary Figure 1B). Interestingly, subsequent analysis showed that miR-200a inhibition specifically lead to an upregulation of c-Jun protein in GFAP^+^ glial cells (Fig. 5b). A small but insignificant number of GFAP^+^ glial cells express a low level of c-Jun (Fig. 5b, c), however, after inhibition of miR-200a there is a significant increase in the number of GFAP^+^ cells that express c-Jun at the protein level **(**Fig. 5b, c). Luciferase reporter experiments confirmed that the axolotl c-Jun 3′-UTR is directly targeted by miR-200a (Fig. 5d) and mutation of the miR-200a seed sequence alleviates that repression (Fig. 5d). Collectively, these experiments confirm that miR-200a represses c-Jun expression in GFAP^+^ glial cells.

### miR-200a inhibition affects glial scar related genes

MicroRNAs are able to regulate expression of dozens, if not hundreds, of genes in multiple pathways to precisely coordinate various cellular processes^78^. To gain a more comprehensive picture of the role of miR-200a in regulating the pro-regenerative glial cell response during axolotl spinal cord regeneration we performed RNA sequencing analysis. Uninjured, control and miR-200a inhibitor electroporated spinal cords were isolated at 4 days post injury, one day after peak miR-200a upregulation and processed for RNA sequencing (Fig. 3a).

**Fig. 6:**
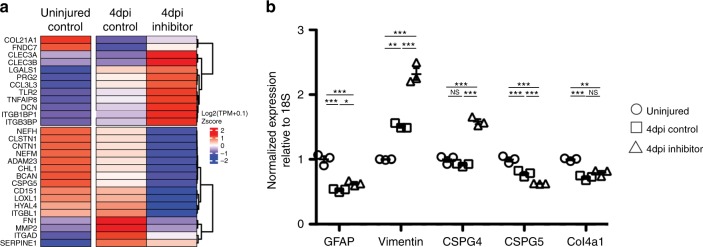
miR-200a inhibition leads to differential expression of genes involved with reactive gliosis and glial scar formation. **a** Heatmap representation of expression values, transcription per million expression values (TPMs) for uninjured, 4 days post injury control and 4 days post injury miR-200a inhibitor electroporated samples were padded (+0.1) and log2 transformed. Mean values across replicates were centered and scaled prior to clustering. **b** qRT-PCR analysis confirms differential expression of reactive gliosis and glial scar related genes after miR-200a inhibition. *p ≤ 0.05, **p ≤ 0.01, ***p ≤ 0.001, N.S. is not significant. Error bars represent ± S.T.D

Analysis of the RNA sequencing data compared the expression profiles at 4 days post injury of control or miR-200a inhibitor treated spinal cords to uninjured spinal cords was carried out (Supplementary Data 1). From these comparisons genes that had fold change in expression greater than 2.0-fold and a significant *p*-value (*p* ≤ 0.05) in one or both comparisons were included in further analysis. This approach identified a cohort of genes that were differentially regulated at 4 days post injury after miR-200a inhibition (Fig. 6a, Supplementary Data 1). A cohort of 462 genes were identified with a greater fold change in the miR-200a inhibitor samples. (δlog2FC > 1). While 153 genes were found to be downregulated in the miR-200a inhibitor treated samples (δlog2FC < -1) (Supplementary Figure 3). From research in mammals a subset of these genes (Fig. 6) are known to be involved with reactive gliosis and glial scar formation (LGALS1, DCN, BCAN, TLR2, CSPG5) and have functions related to extracellular matrix (COL21A1, CNTN1, FN1) and ECM remodeling (ADAM23, LOXL1, HYAL4, MMP2, SERPINE1) as well as cell migration (ITGBL1, ITGAD, CD151 ITGB1BP1, ITGB3BP), axon migration (CHL1, CLSTN1, NEFM/H) and inflammation (TLR2, CCL3L3, TNFAIP2)^79–87^. These data collectively suggest that miR-200a inhibition is sufficient to disrupt expression of genes involved with multiple cellular processes necessary for regeneration.

We used qRT-PCR analysis to confirm and analyze more carefully a subset of genes well documented to be involved with reactive gliosis and glial scar formation. This confirmed that miR-200a inhibition leads to a highly significant increase in vimentin expression and a small but significant increase in GFAP expression (Fig. 6b) compared to 4 days post injury control spinal cords. Remarkably, expression of the chondroitin sulfate proteoglycan, CSPG4, which is consistently seen to be upregulated after spinal cord injury in mammals^88–90^, is specifically upregulated after miR-200a inhibition (Fig. 6b). In situ hybridization was used to further validate the qRT-PCR data, both vimentin and CSPG4 were found to be robustly expressed in glial cells in animals treated with miR-200 inhibitor in comparison to control animals **(**Supplementary Figure 4A, B**)**. These data suggest that inhibition of miR-200a after injury leads to a more reactive phenotype and an overall more axon growth inhibitory microenvironment similar to what is observed in mammalian astrocytes after SCI.

## Discussion

**Fig. 7:**
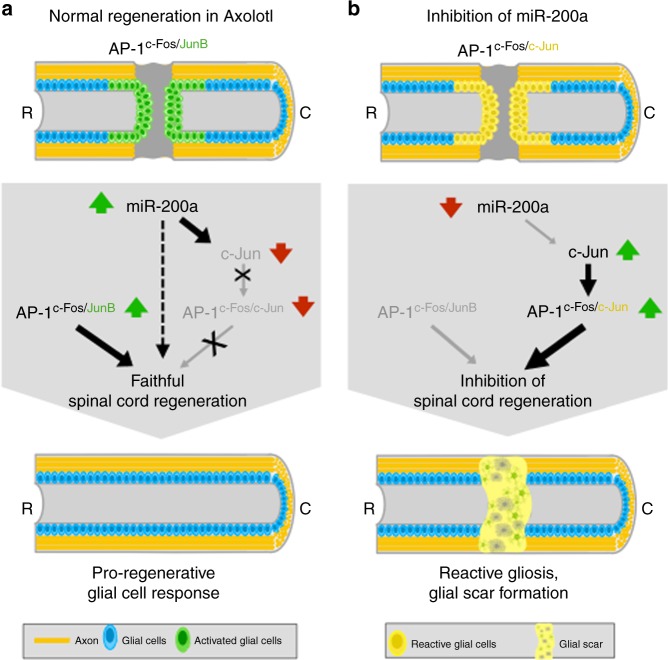
Model of the potential role of AP-1 and miR-200a in spinal cord regeneration. **a** After SCI, axolotl glial cells up-regulate expression of AP-1^cFos/JunB^, which functions to repress GFAP expression and to regulate downstream pathways that direct glial cells to divide, migrate and functionally repair the missing spinal cord tissue. Additionally, miR-200a is upregulated which represses c-Jun expression thereby blocking formation of the canonical AP-1^cFos/cJun^. **b** Inhibition of miR-200a during spinal cord regeneration results in higher levels of GFAP expression, ectopic expression of c-Jun in GFAP^+^ glial cells, upregulation of genes indicative of a glial scar like environment and a failure in axon regeneration

The present study has identified an important role for the non-canonical AP-1^cFos/JunB^ and miR-200a in regulating the pro-regenerative glial cell response to SCI in axolotl (Fig. 7). Our previous work has shown that upregulation of c-Fos in glial cells is essential for proliferation of the glial cells and subsequent axon regeneration^42^. Unlike JUN proteins, which can homodimerize to form AP-1, c-Fos functions as an obligate FOS:JUN heterodimer however the identity of the c-Fos binding partner in axolotl glial cells was unclear^60,91–93^.

Immunofluorescence analysis showed that c-Jun is upregulated in NeuN^+^ neurons but not in GFAP^+^ glial cells after injury to the spinal cord (Fig. 1a). Therefore, pro-regenerative glial cells do not express high levels of the canonical AP-1^cFos/cJun^ during axolotl spinal cord regeneration. Interestingly, the JUN family member JunB was instead upregulated and heterodimerizes with c-Fos in axolotl glial cells (Fig. 1b, c, Supplementary Figure 1A). Studies from mammalian CNS injury models have shown that the canonical AP-1^cFos/cJun^ heterodimer is upregulated and promotes reactive gliosis and glial scar formation. Remarkably, mimicking the mammalian situation by ectopically overexpressing AP-1^cFos/cJun^ in axolotl glial cells led to defects in axon regeneration, indicative of an overall growth inhibitory environment, which could be similar to the glial scar in mammals (Fig. 2d). However, we do note that a small portion of axolotl GFAP glial cells do in fact express c-Jun, these could be cells that are on their way to becoming neurons, as it is known GFAP^+^ glial cells have the potential to differentiate into both glial cells and neurons after injury^69,70^.

In other vertebrates that have the ability to regenerate a functional spinal cord Fos and Jun family members are also differentially regulated after injury. In zebrafish, similar to the axolotl, c-Fos and JunB are upregulated 1 day post injury^94^. In addition, lamprey upregulate c-Fos and JunD within 6 h of injury while c-Jun is upregulated 3 days post injury. It is unclear from these data if JunB is upregulated early or if lamprey use JunD instead of JunB^21^. In the future, it will be interesting to look across species to identify how this complex has evolved in regeneration competent versus regeneration incompetent organisms.

AP-1 is capable of regulating expression of many genes involved with cellular proliferation, migration, cell survival, apoptosis, extracellular matrix and ECM remodeling. Some of these processes are mutually exclusive (i.e., cell survival and apoptosis) and the ability of AP-1 to specifically regulate a subset of genes is highly context dependent^58,64,95^. One factor contributing to the ability of AP-1 to regulate target genes is the combination of FOS and JUN proteins that comprise the heterodimer. Additionally, the composition of AP-1 can partially affect its ability to act as a transcriptional activator or repressor. For example, AP-1^FosB/JunB^ transcriptionally activates *FoxD5b* expression during *Xenopus* neural development while AP-1^cFos/cJun^ acts as a transcriptional repressor^96^. Furthermore, JunB can antagonize the ability of c-Jun to transform non-malignant cells^97^. Indeed, often times JunB and c-Jun signaling directly antagonize one another^57,98^. These observations are consistent with our findings that the canonical AP-1^cFos/cJun^ is a potent transcriptional activator of the axolotl GFAP promoter while AP-1^cFos/JunB^ represses the axolotl GFAP promoter (Fig. 2b).

These experiments highlight the possibility that differential combination of AP-1 subunits could induce very different cellular response to injury. While our luciferase assays show AP-1^cFos/JunB^ does not fully repress luciferase activity compared to the control it is important to realize that while *GFAP* expression is downregulated during axolotl spinal cord regeneration (Fig. 2a) there is still a basal level of GFAP expression suggesting homeostatic levels of promoter activity. Additionally, GFAP expression is controlled by multiple transcription factors including NF-κB and STAT3 therefore it is not surprising that AP-1^cFos/JunB^ does not fully repress the GFAP promoter alone^99^. In the future it will be interesting to identify other factors that synergize with AP-1^cFos/JunB^ to regulate the pro-regenerative glial cell response. Indeed, co-occupancy of AP-1 with key pioneering transcription factors is necessary for efficient chromatin remodeling highlighting a possible role for AP-1^cFos/JunB^ in aiding to remodel the chromatin landscape to a more permissive environment to allow for later waves of gene expression that promote the pro-regenerative glial cell response^100,101^.

Our data support a model where pro-regenerative glial cells upregulate AP-1^cFos/JunB^; however, the mechanism by which c-Jun expression is repressed was not clear. Our lab and others have shown that microRNAs play a critical role in fine-tuning the genetic programs necessary for tissue regeneration^10,71–76^. We have identified miR-200a as a microRNA that regulates levels of c-Jun expression in GFAP^+^ glial cell in axolotl. miR-200a is specifically expressed in glial cells adjacent to the central canal and is differentially expressed during spinal cord regeneration (Fig. 3a, b). Importantly, inhibition of miR-200a led to an increase in *c-Jun* transcript abundance and co-expression of c-Jun in GFAP^+^ glial cells (Fig. 4). MicroRNA mediated repression does work in a binary fashion, which explains why GFAP^+^ glial cells in homeostatic conditions do in fact express a low basal level of c-Jun. However it is the upregulation of miR-200a and subsequent repression of c-Jun that is essential to ensure that the levels of the c-Jun protein are kept low, which facilitates a preferential heterodimerization event to occur between the upregulated JunB and c-Fos after injury to the spinal cord. Inhibition of miR-200a blocks spinal cord regeneration (Fig. 3d, Supplementary Figure 2) similar to the phenotype observed with AP-1^cFos/cJun^ overexpression (Fig. 2d). However, miR-200a inhibition resulted in a more severe phenotype than overexpression of AP-1^cFos/cJun^, this may be due to technical reasons. The efficacy of introducing a small molecule inhibitor into the glial cells is far higher than the efficacy of overexpressing two plasmids in these cells. In addition, when we overexpress c-Jun in the glial cells it must compete with the endogenous JunB for binding to c-Fos and hence may result in a less severe phenotype. This does not rule out the possibility that other downstream targets of miR-200a are also affected and are necessary for functional spinal cord regeneration.

In this study, we used chemically synthesized inhibitors to knock down miR-200a levels rather than using a knockout strategy like CRISPR which has been shown to work effectively in axolotl^102,103^. In the future, it will be interesting to determine if a conditional CRISPR knockout of miR-200a in glial cells leads to complete block of regeneration, however the technology is not yet available in axolotl to carry out this experiment. There are several reasons for choosing a knockdown approach, other studies in zebrafish have shown that, in some cases, knockouts do not give a phenotype due to compensation by other related genes and this could easily occur with microRNA genes as there are several very similar family members^104^. More recent work in mouse ES cells and human cells has identified large deletions and complex rearrangement of genomic information as a result of gene knock-out using CRISPR technology^105^. These bodies of work suggest that a less severe knockdown strategy may in fact reveal more about the underlying biology especially in relation to regeneration where we want the genes to be present during development and only interfere with their function during the regeneration process.

While we have identified c-Jun as one miR-200a target, miRNA’s are able to target dozens if not hundreds of genes to orchestrate multiple cellular pathways. Therefore we performed RNA sequencing of spinal cord tissue from uninjured spinal cords and regenerating spinal cords microdissected from 500 micrometers rostral and 300 micrometers caudal to the lesion. This area corresponds to the area from which glial cells contribute to regeneration^42^. While this method does not specifically isolate only the glial cells it allows us to identify gene expression changes resulting from non-cell autonomous affects of miR-200a inhibition and facilitate the identification of molecules that could contribute to the lesion microenvironment. We cannot rule out that mRNA present in the severed axons also contributes to the RNA sequencing data set and in fact the potential role of mRNAs from axons in regeneration may be very important to investigate in future studies. However no genes specific to muscle or skin were identified in the RNA seq data, suggesting that the tissue composition is mainly spinal cord. Hundreds of genes were differentially regulated at 4 days post injury after miR-200a inhibition compared to uninjured spinal cords (Table S1, Supplementary Figure 3). Further analysis identified a handful of genes known to be involved in reactive gliosis and glial scar formation to be upregulated after inhibitor treatment, this we confirmed by qRT-PCR (Fig. 6b). Although the axolotl is a very powerful research organism for studying regeneration, one limitation still faced is the paucity of antibodies available specific to axolotl proteins. However we could confirm upregulation of some of these genes at the mRNA level and see that they are expressed in the glial cells adjacent to the injury site (Supplementary Figure 4). This data suggests that miR-200a is necessary to inhibit a reactive gliosis circuitry from being activated after injury in axolotl glial cells. In addition many of the genes found to be differentially regulated during axolotl regeneration are genes known to be important for spinal cord regeneration in fish and other salamanders, suggesting a high degree of pathway conservation across species (Fig. 6)^12,106–109^. Interestingly, bioinformatics analysis of the promoter regions of GFAP, Vimentin, CSPG4 and CSPG5 using PROMO revealed multiple DNA binding motifs for c-Fos, JunB and c-Jun, further supporting our hypothesis that these factors could differentially regulate these gene promoters during spinal cord regeneration (Supplementary Figure 5).

Collectively, our work identifies an important role for the c-Fos:JunB heterodimer in activation of glial cells after spinal cord injury to guide them to a regenerative state **(**Fig. 7**)**. It also identifies an important molecular difference in a highly conserved early response heterodimer in axolotls versus mammalian glial cells.

## Methods

### Animal handling, spinal cord injury and electroporation

All axolotls used in these experiments were obtained from the Genetic Stock Center at the University of Kentucky, bred at the University of Minnesota in accordance with IACUC protocol No. 1710-35242A, or bred the Marine Biological Laboratory in compliance with IACUC protocol number 18–49. All animal experiments were carried out in compliance of USA animal welfare legislation. In all experiments the white strain of *Ambystoma mexicanum* was used, all animals were 3–5 cms in length, a size at which sex is not yet identifiable. Prior to all in vivo experiments animals (3–5 cm) were anesthetized in 0.01% p-amino benzocaine (Sigma). Spinal cord ablations were performed as previously described^10,11^. Briefly, animals were anesthetized, the skin and muscle above the spinal cord was peeled back using fine forceps. Then a piece of spinal cord, approximately the length of 1 muscle bundle, was transected using small surgical scissors and gently removed. The skin flap was replaced and the animal was placed back into water to recover. For miR-200a inhibition experiments, a chemically synthesized miR-200a or scrambled control inhibitor (Dharmacon) were diluted to 20uM in PBS + 1% Fast Green and microinjected into the central canal of axolotls and were electroporated (5 square pulses, 50 ms, 50 V using an ECM830 Electro Square Porator BTM Harvard Apparatus) twice. For overexpression experiments plasmids were diluted to 1–1.5 μg/μL in PBS + 1% Fast Green and animals were injected and electroporated as described above.

### Immunofluorescence

Tissue was harvested at the indicated time points and were fixed and processed for cryosectioning as previously described^11^.

For IHC, tails were sectioned at 20 μm using a Leica CM1850 cryostat. The following primary antibodies were used for immunofluorescent staining: c-Jun (1:100, Cell Signaling), GFAP (1:100 Chemicon) or NeuN (1:100 Chemicon). All sections were incubated in boiling 10 mM sodium citrate buffer + 0.1% Tween 20 for 10 min prior to standard IHC protocol^11^. After secondary incubation the slides were washed four times with PBSTx and embedded with 80% glycerol and imaged using an inverted Leica DMI 6000B fluorescent microscope.

Double positive GFAP^+^/cJun^+^ glial cells were quantified by counting the number of glial cells expressing nuclear c-Jun that were also GFAP immunoreactive. The percent double positive glial cell was calculated by the number of GFAP^+^/cJun^+^ cells divided by the total number of GFAP^+^ glial cells.

### Whole mount immunofluorescence

Tails were harvested at 7 days post injury and fixed in 4% PFA. Whole mount β-III tubulin staining was performed as previously described^10,11^ with one minor modification. Secondary antibody (anti-mouse 568 Invitrogen) staining was left overnight at 4 °C. After washing off excess secondary, tails were gradually stepped into methanol by incubating with 25%, 50%, 75% then 100% methanol:PBSTx solution and then stored at –20 °C until imaged. Prior to imaging, the tails were cleared using 1:2 solution of benzyl alcohol and benzyl benzoate (Sigma) for 20 min and mounted onto a cover slip using BABB as the mounting medium.

### EdU staining

Control and miR-200a inhibitor electroporated animals were injected with 5-ethynyl-2´-deoxyuridine (EdU) at a final concentration of 0.3 μg/μL. For glial cell proliferation, EdU was injected 3 days post injury and tissue was harvested 24 h later. For neuronal differentiation, EdU was injected on the day of injury and tissue was harvested 5 days later. Samples were prepared for EdU and antibody staining as described above. EdU staining was carried out on frozen cross-sections. EdU incorporation was detected using the Click-iT EdU Alexa Fluor 555 Imaging kit (Molecular Probes) prior to GFAP or NeuN staining.

### Acid fuschin orange G staining

AFOG staining was performed on cross-sections and paraffin sections as described previously^11^.

### qRT-PCR

The spinal cords 500 μm rostral and 300 μm caudal to the lesion from 7–10 control or miR-200a inhibitor electroporated animals were microdissected and pooled for each biological replicate. Total RNA was isolated using Trizol according to the manufacturer. Subsequent cDNA was synthesized from 1 μg of DNaseI (NEB) treated RNA using either High Capacity cDNA Reverse Transcription kit (Applied Biosystems) or miScript II RT kit (Qiagen). The qRT-PCR was carried out using LightCycler 480 SYBR Green I Master (Roche). MicroRNA qRT-PCR was carried out with custom designed primers to conserved miRNAs (Qiagen) and custom primers from IDT were used to quantify axolotl mRNAs (5′–3′):

18S_F: CGGCTTAATTTGACTCAACACG

18S_R: TTAGCATGCCAGAGTCTCGTTC

cFos_F: TCCCTCTACACCTCCGAC

cFos_R: AAAGCGTCCGATTCAGGG

cJun_F: CTCTGCCCCAAGAATGTGAC

cJun_R: GAAGTTGCTGAGGTTGGCAT

JunB_F: CTCCTTCCTGCCTGGCTATG

JunB_R: ACTGTCCGAGCCAAAGTAGC

GFAP_F: ACAGAGCCTAAACAGTGATG

GFAP_R: GTCTTTAAGGTTCCGGATGT

Vimentin_F: AACACTCTCCAGTCTTTCAG

Vimentin_R: TCTTCGTCGTGTAGTTTCTT

CSPG4_F: ATTCCATTACCCCACCTAGT

CSPG4_R: AGCTGCCCCTCATTAATATG

CSPG5_F: CATGATGACCGTTTTCTTCG

CSPG5_R: GATGGTGGACAGAGAAAAGT

Col4a1_F: GTGGCTATCTCTCTGGATTG

Col4a1_R: CCATGGCACTCAATAAATGG

### microRNA and mRNA in situ hybridization

Samples were fixed in fresh 4% PFA at 4 °C overnight. The following day they were washed 3 times for 10 min in PBS. Then they were gradually stepped into 100% methanol and stored at –20 °C. The samples were gradually stepped back into PBS and processed for cryosectioning, as described above. To remove OCT after sectioning the slides were washed 3 times for 10 min in PBSTw before being incubated with Protinase K (2 μg/μL) for 10 min. The slides were rinsed once and then washed for 2 min in a glycine solution (2 mg/mL). Then slides were rinsed 3 times with PBSTw and incubated in a 1:1 solution of hybridization buffer:PBSTw for 10 min. Following that the slides were incubated in hybridization buffer plus yeast tRNA extract for 3 h at room temperature. After the pre-hybridization step miR-200a LNA probe (Exicon) was added to the slides at a final concentration of 40 nM. Slides were hybridized over night at 53 °C. The following day slides were washed in 5x SSC buffer for 10 min then 0.2% SSC buffer for one hour. Both washes were carried out at 60 °C. The slides were then stepped back into PBSTw and washed one final time in PBSTw for 10’. Then slides were blocked in PBSTw + 2% BSA + 2% sheep serum for 1 h at room temperature and anti-DIG antibody (Roche) was diluted 1:1000 in blocking buffer for 2 h. Slides were then rinsed 2 times in PBSTw and washed 3 times for 10 min in PBSTw before being incubated in fresh AP buffer 3 times for 5 min. After the final wash BM Purple was added and slides were checked often for color development. Finally, upon completion of the reaction sections were fixed in 4% PFA for 10 min, rinsed 3 times in PBSTw then embedded with 80% glycerol and imaged using an Olympus BX40 inverted microscope.

### JunB probe synthesis

Axolotl specific JunB probes were created by PCR amplification with the addition of the T7 and Sp6 promoter into the PCR primers:

T7 JunB ISH For 5′-AGAtaatacgactcactatagggAATGTGCCGTGCAGCGGATA-3′

Sp6 JunB ISH Rev 5′-AGAtatttaggtgacactatagAAGAGGTAGAGGGAGCCCAGTC-3′

The resulting PCR product was used to synthesize in situ probe by the addition of DIG-labeled UTP (Roche) plus the appropriate RNA Polymyerase T7 or Sp6 (NEB). Probes were purified with RNA Clean Up kit (Qiagen) and resuspended in 100 μL of hybridization buffer.

Primers for other genes (5′–3′):

Vimentin ISH Forward ACAAGTCAAAGTTCGCTGAT

Vimentin ISH Reverse CCATCTCTGGTCTCAACAGT

NG2 ISH Forward CTTACTGTCGACGAGGAGAC

NG2 ISH Reverse TCGGGCTGTTGTACTATCTT

Galectin-1 Forward TAGGGGTCATGTGACTTTTC

Galectin-1 Reverse AGGCAACTAGTCCAGTTTGA

The PCR fragment was subcloned into pGemTEasy vector. The plasmid was then linearized and used to synthesize in situ probe by the addition of DIG-labeled UTP (Roche) plus the appropriate RNA Polymyerase T7 or Sp6 (NEB). Probes were purified with RNA Clean Up kit (Qiagen) and resuspended in 100 μL of hybridization buffer.

### mRNA in situ hybridization

Samples were prepared as described above. After sectioning slides were incubated in 70 °C PBS for 15 min to remove OCT. Then the slides were incubated in 1:1 PBS:EtOH for 5 min before being washed in 100% EtOH for 5 min. Then the slides were incubated in Xylene for 30 min before being washed twice in 100% EtOH. After washing, the slides were transferred to 100% MeOH for 5 min and gradually stepped into PBST. Then slides were incubated in 1:1 PBST:Hyb for 10 min and pre-hybridized for 30 min. JunB probes were diluted into hybridization buffer and slides were allowed to hybridize overnight at 55 °C. The following day the slides were washed 3 times in Wash Buffer (50% Formamide, 5x SSC and 0.1% Tween) for 30 min each, once in 1:1 Wash Buffer:PBST for 30 min and once in PBST at 55 °C. Slides were rinsed in room temperature PBST 3 times for 5 min each before blocking buffer was added (2% goat serum, 2% BST in PBSTx) for 1 h. Anti-DIG F_AB_ (Roche) was diluted 1:1000 in blocking buffer and slides were incubated for at least 1 h. Slides were washed 3 times for 10 min each before addition of fresh AP Buffer for at least 10 min. Finally slides were incublated in BM Purple (Roche) until colored reaction was observed. The reaction was stopped by several quick rinses in PBS and slides were fixed in 4% PFA for 10 min. Slides were mounted in 80% glycerol and images were taken with a BX40 Olympus Inverted microscope.

### Cloning and plasmids

The full-length axolotl JunB and axolotl cJun 3′-UTR were cloned using rapid amplification of cDNA ends (Clontech) as per the manufacturer’s instructions.

JunB 5′ GSP 1: GCTGCACCACTGTCCGAGCCAAAGT-3′

JunB 5′ NGSP 1: TGGGTCAGTGAGGTTAAGGGCCAAGC-3′

JunB 3′ GSP 1: CCTCAACCCCACTACTCCACCTCGG-5′

JunB 3′ NGSP 1: GACCAAGAGCGCATTAAGGTGGAGCG-3′

cJun 3′ GSP 1: GAACCGCATCGCCGCCTCCAAGTG-3′

cJun 3′ NGSP 1: GCAGAACTCGGAGCTGGCTTCCACG-3′

The coding sequences of axolotl c-Fos and c-Jun were cloned based on Trinity assembled RNA sequencing contigs from axolotl-omics.org. The coding sequence of axolotl JunB was amplified using primers based on the full-length transcript from our RACE experiments. PCR fragments for c-Fos, c-Jun and JunB were digested with the indicated restriction enzymes and ligated into the pCMV:GFP expression vector (Clontech). Restriction fragments were ligated together using T4 DNA Ligase (NEB) overnight at 4 °C and heat shock transformed into DH5α competent *E*. *coli* (Promega)

cFos For Axolomics NheI ATTGCTAGCACCATGTTCCAGGGCTTCTCGGG

cFos Rev Axolomics SacII ATCCCGCGGCAGAGCAAGCAAAGTAGGCG

cJun For XhoI ATTCTCGAGACCATGGAGCCTACGTTCTACG

cJun Rev SalI ATTGTCGACACATGAACGTCTGCAGCTGCTG

JunB For NheI ATTGCTAGCACCATGTGCACCAAGATGGACG

JunB Rev SacII ATTCCGCGGAAAGGGCTGCATCTTGGCA

Sequences for the human versions of c-Fos (#70382), c-Jun (#70398) and JunB (#29687) were cloned from the indicated Addgene plasmids using the following primers (5′–3′) and subcloned into pCMV:GFP (Clontech):

cFos FL Hs For SalI TATGTCGACACCATGACTGCAAAGATGGAAACGA

cFos FL Hs Rev BamHI ACTGGATCCAAATGTTTGCAACTGCTGCGTTAG

cJun FL Hs For SalI TATGTCGACACCATGACTGCAAAGATGGAAACGA

cJun FL Hs Rev BamHI ACTGGATCCAAATGTTTGCAACTGCTGCGTTAG

JunB FL Hs For SalI TATGTCGACACCATGTGCACTAAAATGGAACAGCC

JunB FL Hs Rev BamHI ACTGGATCCGAAGGCGTGTCCCTTGAC

For 3’ UTR luciferase experiments, primers were designed to amplify the cJun 3’ UTR based off our RACE sequences. All the 3’ UTRs were amplified with a 5′ SpeI and 3′ HindIII restriction site.

cJun 3′-UTR Luc For ATAACTAGTGGAGGGAAGCGCGG

cJun 3′-UTR Luc Rev AGCAAGCTTGTTTGTAGTTTAGTTTGTAATAC

cJun 3′-UTR SDM Rev2 GATCTGTTTGAATTCACCAAGAACTGCAT

cJun 3′-UTR SDM For 2 GTTCTTGGTGAATTCAAACAGATCTCGC

The PCR fragments and pMiR Report (Life Technologies) were digested with SpeI and HindIII (NEB) and the fragments were ligated over night at 4 °C with T4 DNA Ligase (NEB) and heat shock transformed into DH5α competent E. coli (Promega).

### Cloning of the axolotl GFAP promoter

Genomic DNA was isolated from tails of 2–3 cm axolotls using Jetflex Genomic DNA Purification kit (Invitrogen). Following purification of genomic DNA, we used GenomeWalker Universal kit (Clontech) to determine the DNA sequence upstream of the GFAP transcription start site. Two rounds of “walking” were performed using the below primers (5′–3′):

GFAP Promoter GSP1 TTTCCCAACTTCAGCCTCGCAAGACTC

GFAP Promoter NGSP1 ACTTTTGAGGAGGGCCGTTAGAGAAC

GFAP Promoter GSP2 TTCACTGTGGCGTCATGTGGATCGGTAACC

GFAP Promoter NGSP2 TTCCAGCACACTCTGCGTCCCTTTGTTTGC

Distinct PCR bands TA cloned into pGEM-T EZ (Promega) and inserts were sequenced. Based on this analysis we designed primers to amplify ~1.3 kb of the axolotl GFAP promoter using Phusion High Fidelity polymerase (NEB) (5′–3′):

pAxGFAP For 2 XhoI ATACTCGAGTACCTGGCATTGACATTATCTGGTC

pAxGFAP Rev1 HindIII ATAAAGCTTTTTCAGAGTTTCCCAACTTCAGCCT

The resulting PCR product was ligated as a XhoI and HindIII fragment into pGL3 Enhancer luciferase reporter plasmid (Invitrogen).

### Luciferase experiments

3’ UTR luciferase experiments

B35 neural progenitor cells were plated in a 96 well plate (Celltreat Scientific Products) at a concentration of 1.0 × 10^5^ cells/mL and allowed to adhere overnight. The next day cells were co-transfected with axolotl c-Jun 3’ UTR luciferase reporter, β-Galatosidase internal control and 100 nM of miR-200a or control mimic (Qiagen) Lipofectamine 3000 (Invitrogen). After 48 h luciferase activity was determined using Dual Light Reporter system (Thermo) according to the manufacturers protocol.

### GFAP promoter luciferase assays

B35, neural progenitor cells were co-transfected with pGFAP: Luciferase, β-Galatosidase internal control, axolotl c-Jun and/or JunB subcloned into pCMV:GFP plasmid (Clontech) with Lipofectamine 3000. After 48 h luciferase activity was determined using Dual Light Reporter system (Thermo) according to the manufacturer’s protocol.

The similar experiment was carried out using the axolotl GFAP promoter.

### BioID experiments

The axolotl JunB ORF was subcloned into the EcoRV and BamHI sites of BioID Myc tag vector (Addgene #35700). (5′–3′):

JunB_BioID_Myc_For ATAGATATCTACCATGTGCACCAAGATGGAG

JunB_BioID_Myc_Rev AGGGGATCCTCAAAAGGGCTGCATCTTG

A total of 293 cells were plated in 24-well plate (1 × 10^5^ cells per well) and transfected with either empty BioID + c-Fos, JunB-BioID alone or JunB-BioID + cFos. After 24 h biotin (50 μM) was added to the cell culture media and allowed to incubate for an additional 24 h. Finally, cells were lysed in boiling RIPA buffer and sonicated to lyse cells and sheer DNA. Then 50 μL of pre-washed Dynabeads MyOne Streptavidin T1 (ThermoFischer) was added per lysate and allowed to incubate at 4 °C. The following day the beads were washed 4 times in RIPA buffer and bound proteins were released from beads by addition of 25 μL of LSB, an excess of biotin and incubated at 95 °C for 5 min.

### Western blot analysis

Western blot was performed as previously described^11^. Anti-c-Fos antibody (Millipore) was diluted 1:500 in 5% milk in Tris Buffered Saline + 0.1% Tween and incubated at 4 °C overnight. Anti-Rabbit DyLight 68o (Cell Signaling) was diluted 1:10000 in blocking buffer for 1 h. Following 4 TBST washes signal was detected using a Licor Odyssey CLx.

### RNA sequencing sample preparation

For RNA sequencing animals (3–5 cm) were electroporated with miR-200a inhibitor or control inhibitor and a spinal cord trasection was performed, as described above. Animals were electroporated on the day of injury and 2 days post injury. Spinal cords 500 μm rostral and 300 μm caudal to the lesion were microdissected at 4 days post injury for RNA extraction using TRIzol (Thermo Scientific) according to the maufacturers protocol. Each sample (Uninjured, 4 days post inury Control and 4 ddays post injury Inhibitor) consisted of 6 pooled spinal cords and samples were submitted in biological triplicates. Library preparation and RNA sequencing was performed by the Genomics Center at the University of Minnesota.

### RNA-seq data analysis

RNA-seq data were comprised of 2 × 126 bp paired-end reads. The RNA-seq reads from all samples were combined and then assembled by using Trinity^110^ (v2.3.2) with default parameters. We used cd-hit-est (v4.6)^111^ with parameter “-c 1” to remove shorter contigs with 100% identity with aligned longer contigs. These non-redundant contigs were mapped to Ensembl human proteins (v85) by BLASTX (v2.2.18). Contigs were assigned to human proteins by taking the best BLASTX hit with E-value < 10-5.

We used Bowtie (v0.12.1)^112^ to map the reads against all non-redundant contigs. Quantification of each contig was performed by RSEM v1.1.6^113^. RSEM employs an expectation maximization algorithm, so that for reads that match to multiple contigs, RSEM assigns a fraction of each read to each contig based on estimated abundances of contigs based on unique reads^113^.

For each sample, the expected fragment counts for each contig (as computed by RSEM), were then converted to comparative transcript counts by summing the fragment counts of contigs mapped to the same transcript. Similarly, gene-level counts were obtained by summing the fragment counts of transcripts that were annotated with the same gene symbol. Relative abundances, in terms of transcripts per million (TPM), for genes were computed by first normalizing each gene’s fragment count by the sum of the effective lengths (weighted average of contigs’ length based on contigs’ abundance) of the contigs mapped to that gene and then scaling the resulting values such that they summed to one million over all genes, as described previously^114^. To identify differentially expressed genes, the normalized counts from RSEM were imported into DESeq2 (version 1.16.1)^115^ as integers to determine moderated estimates of Log2 fold changes and Benjamini-Hochberg adjusted *p*-values between conditions.

### Statistical analyses

All results are presented as mean + /− s.t.d. unless otherwise stated. Analyses were performed using Microsoft Excel or GraphPad Prism. Data set means were compared using ANOVA for three or more tests with a Dunnett’s or Tukey’s post hoc analysis. When two groups were compared a Students *t-*test was used. Differences between groups was considered significant at three different levels (*p* values of *p < 0.05, **p < 0.01 and ***p < 0.001) and are indicated in the figure legends.

### Reporting summary

Further information on experimental design is available in the Nature Research Reporting Summary linked to this article.

## Supplementary information

Supplementary Data 1

Description of Additional Supplementary Files

Supplementary Information

Reporting Summary

## Data Availability

The authors declare that all data supporting the findings of this study are available within the article and its Supplementary Information files or from the corresponding author upon reasonable request. The RNA seq data has been deposited in the public GEO database with the accession number GSE122939.
