# Supplementary material for: AP-1cFos/JunB/miR-200a regulate the pro-regenerative glial cell response during axolotl spinal cord regeneration
Source: Commun Biol. 2019 Mar 6;2:91. doi: 10.1038/s42003-019-0335-4 (PMC6403268; doi:10.1038/s42003-019-0335-4)
Supplement: Supplementary file 2 — Description of Additional Supplementary Files [file 42003_2019_335_MOESM2_ESM.pdf]

Description of Additional Supplementary Files

File Name: Supplementary Data 1

Description: Table of all transcripts per million (TPMs) from all RNA seq data contained in the manuscript.
