# Supplementary material for: AP-1cFos/JunB/miR-200a regulate the pro-regenerative glial cell response during axolotl spinal cord regeneration
Source: Commun Biol. 2019 Mar 6;2:91. doi: 10.1038/s42003-019-0335-4 (PMC6403268; doi:10.1038/s42003-019-0335-4)
Supplement: Supplementary file 3 — Supplementary Information [file 42003_2019_335_MOESM3_ESM.pdf]

Sabin et al Supplementary Data

Supplementary Figure 1

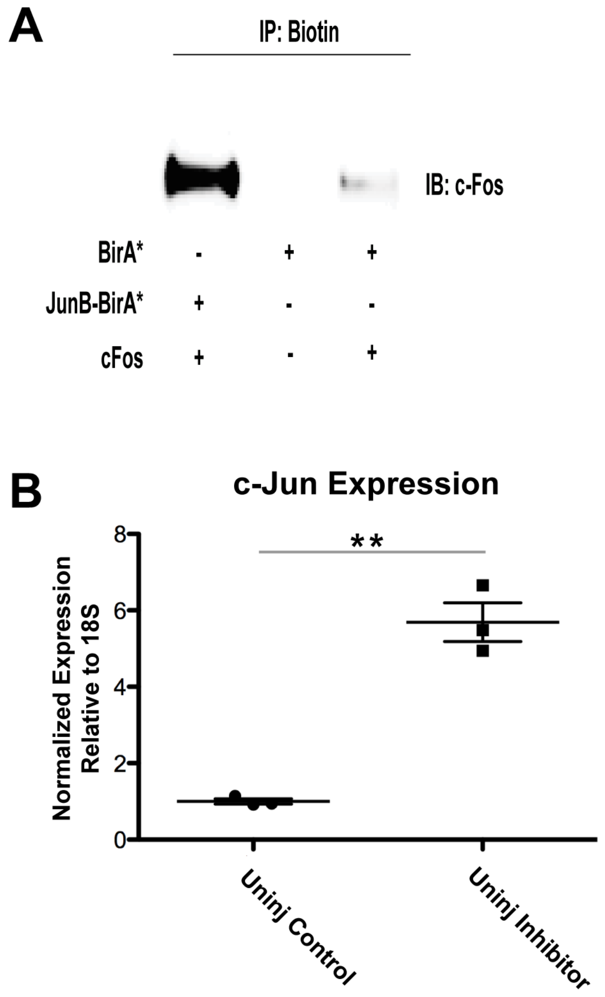

Supplemental Figure 1: Axolotl c-Fos and JunB biochemically interact. (A) The axolotl JunB open reading frame was subcloned into the BioID vector. 293 cells were transfected with JunB-BioID+c-Fos, axolotl c-Fos or empty BioID. Subsequent Western blot analysis shows there is only robust c-Fos pull down in the presence of JunB-BioID and not empty BioID, confirming specific biochemical interaction (n=2).

(B) c-Jun is up-regulated in uninjured spinal cords treated with miR-200a inhibitor. $^{**}p \leq 0.01$. Error bars represent ±S.T.D.

Supplementary Figure 2

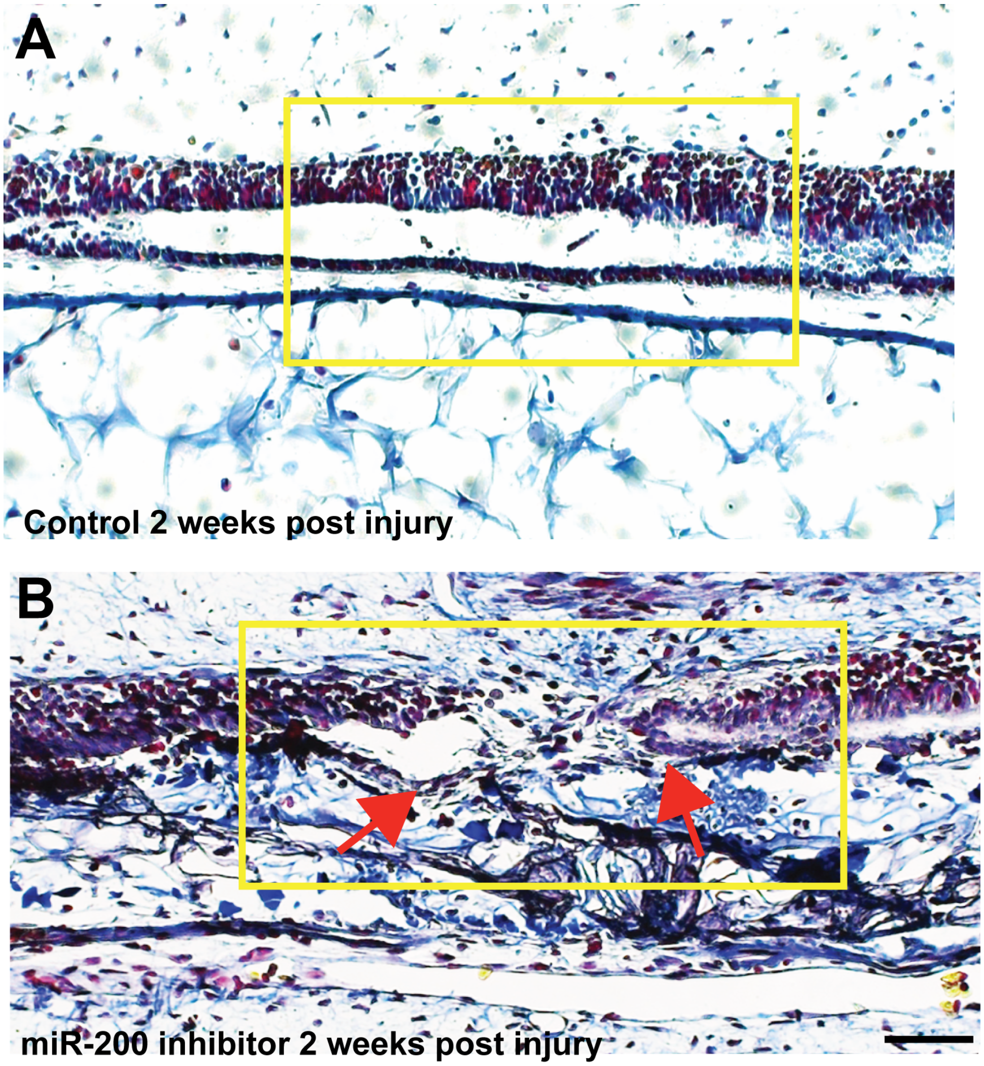

Supplemental Figure 2: Histological staining of control versus inhibitor animals.
(A) Longitudinal section of spinal cord two weeks post injury. Yellow box indicates the site of original injury. (B) miR-200 inhibitor treated animals two weeks post injury. Rostral and caudal sides of the neural tube indicated by red arrows have failed to reconnect. N= 6 control, n= 8 miR-200 inhibitor, Scale bar = 100µm.

Supplementary Figure 3

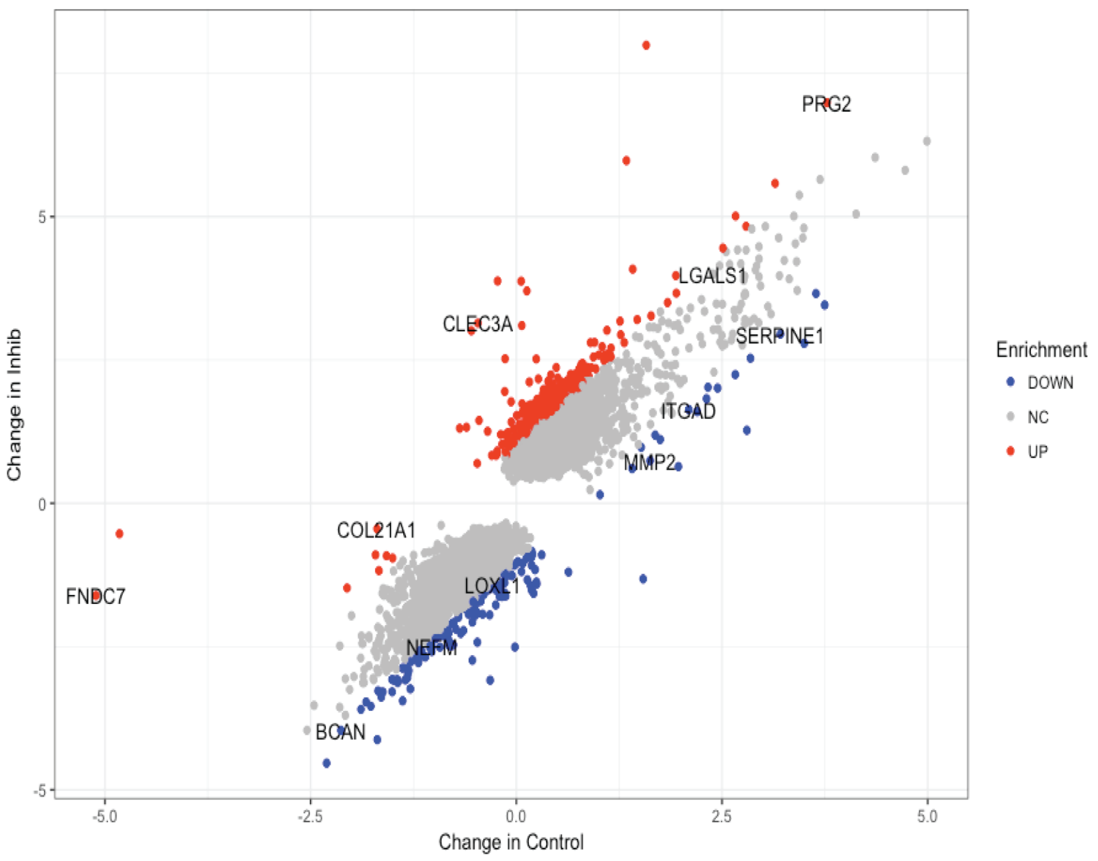

Supplemental Figure 3: miR-200a inhibition leads to differential expression of a cohort of genes.
Scatter plot representing log2 fold changes in gene expression at 4 days post injury in control versus miR-200a inhibitor electroporated spinal cords relative to the uninjured animals. Genes with a mean count greater than 50 and a Benjamini-Hochberg adjusted p-value less than 0.1 in one or both experiments are shown. Genes with expression changes greater than 2-fold (log2 > 1 or log2 < -1) between experiments are colored red or blue for up and down regulated respectively. Labeled points represent a subset of genes involved with reactive gliosis, glial scar formation, ECM, ECM remodeling and axon migration.

Supplementary Figure 4

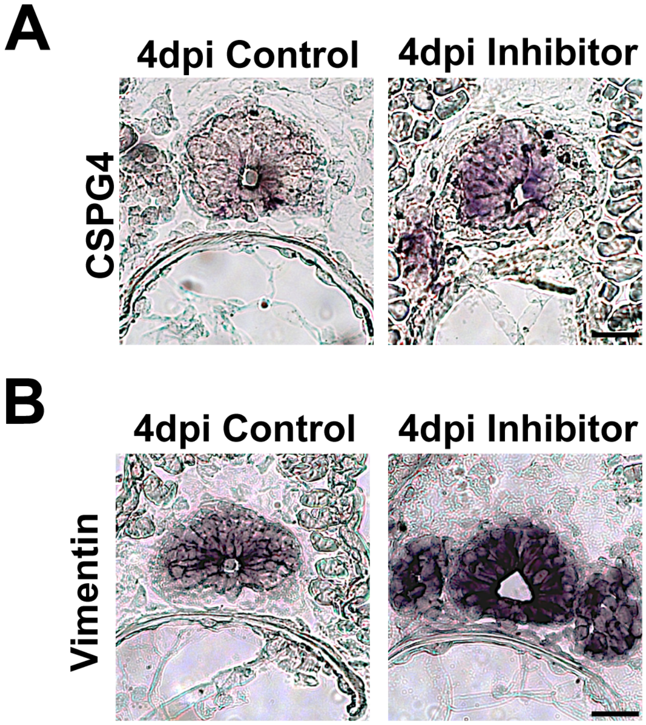

Supplemental Figure 4: In situ hybridization of genes up-regulated in miR-200a inhibitor treated animals. (A) Cross-section of regeneration spinal cord, CSP$ levels are increased in when miR-200a is inhibited. (B) Vimentin levels are also increased in glial cells during regeneration when miR-200a is inhibited.

Supplementary Figure 5

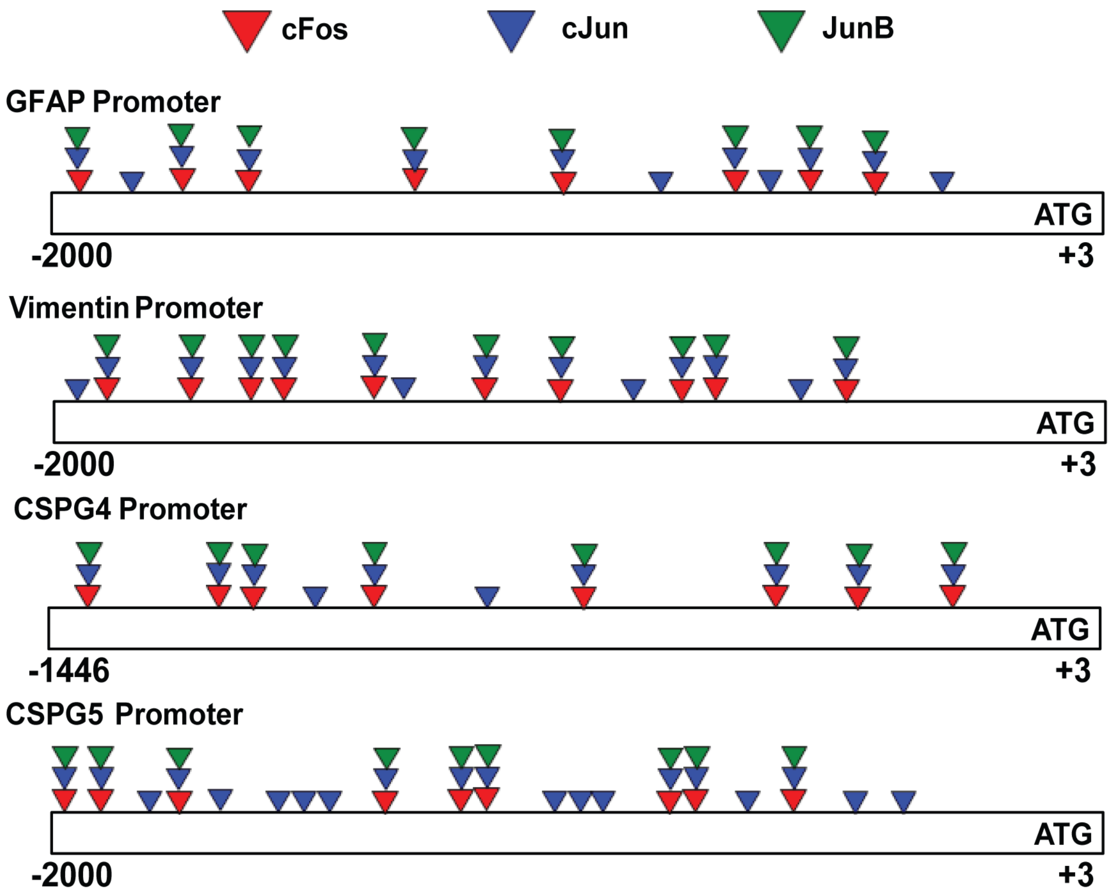

Supplemental Figure 5: Schematic representation of c-Fos, c-Jun and JunB binding motifs in the promoter regions of reactive gliosis and glial scar related genes. Distribution of AP-1 subunit binding motifs in upstream regulatory elements of the axolotl GFAP, Vimentin, CSPG4 and CSPG5 promoters as determined by JASPR. Red triangles represent c-Fos binding motifs, blue triangles represent c-Jun binding motifs and green triangles represent JunB binding motif.
