# Supplementary material for: AP-1cFos/JunB/miR-200a regulate the pro-regenerative glial cell response during axolotl spinal cord regeneration
Source: Commun Biol. 2019 Mar 6;2:91. doi: 10.1038/s42003-019-0335-4 (PMC6403268; doi:10.1038/s42003-019-0335-4)
Supplement: Supplementary file 4 — Reporting Summary [file 42003_2019_335_MOESM4_ESM.pdf]

natureresearch

Corresponding author(s): Karen Echeverri

Reporting Summary

Nature Research wishes to improve the reproducibility of the work that we publish. This form provides structure for consistency and transparency in reporting. For further information on Nature Research policies, see Authors & Referees and the Editorial Policy Checklist.

Statistical parameters

When statistical analyses are reported, confirm that the following items are present in the relevant location (e.g. figure legend, table legend, main text, or Methods section).

n/a	Confirmed	
☐	☒	The exact sample size (*n*) for each experimental group/condition, given as a discrete number and unit of measurement
☐	☒	An indication of whether measurements were taken from distinct samples or whether the same sample was measured repeatedly
☐	☒	The statistical test(s) used AND whether they are one- or two-sided<br>*Only common tests should be described solely by name; describe more complex techniques in the Methods section.*
☐	☒	A description of all covariates tested
☐	☒	A description of any assumptions or corrections, such as tests of normality and adjustment for multiple comparisons
☐	☒	A full description of the statistics including central tendency (e.g. means) or other basic estimates (e.g. regression coefficient) AND variation (e.g. standard deviation) or associated estimates of uncertainty (e.g. confidence intervals)
☐	☒	For null hypothesis testing, the test statistic (e.g. *F*, *t*, *r*) with confidence intervals, effect sizes, degrees of freedom and *P* value noted<br>*Give P values as exact values whenever suitable.*
☒	☐	For Bayesian analysis, information on the choice of priors and Markov chain Monte Carlo settings
☐	☒	For hierarchical and complex designs, identification of the appropriate level for tests and full reporting of outcomes
☒	☐	Estimates of effect sizes (e.g. Cohen's *d*, Pearson's *r*), indicating how they were calculated
☐	☒	Clearly defined error bars<br>*State explicitly what error bars represent (e.g. SD, SE, CI)*

Our web collection on statistics for biologists may be useful.

Software and code

Policy information about availability of computer code

Data collection	*Provide a description of all commercial, open source and custom code used to collect the data in this study, specifying the version used OR state that no software was used.*
Data analysis	Open source software Fiji was used.

For manuscripts utilizing custom algorithms or software that are central to the research but not yet described in published literature, software must be made available to editors/reviewers upon request. We strongly encourage code deposition in a community repository (e.g. GitHub). See the Nature Research guidelines for submitting code & software for further information.

Data

Policy information about availability of data

All manuscripts must include a data availability statement. This statement should provide the following information, where applicable:

- Accession codes, unique identifiers, or web links for publicly available datasets
- A list of figures that have associated raw data
- A description of any restrictions on data availability

The authors declare that all data supporting the findings of this study are available within the article and its Supplementary Information files or from the corresponding author upon reasonable request. The RNA seq data has been deposited in the public GEO database with the accession number GSEXXXXX.

Field-specific reporting

Please select the best fit for your research. If you are not sure, read the appropriate sections before making your selection.

☒ Life sciences ☐ Behavioural & social sciences ☐ Ecological, evolutionary & environmental sciences

For a reference copy of the document with all sections, see nature.com/authors/policies/ReportingSummary-flat.pdf

Life sciences study design

All studies must disclose on these points even when the disclosure is negative.

Sample size	These experiments mainly use live salamander animals, a sample size of 10 animals is a general standard in the developmental biology community.
Data exclusions	No data was excluded
Replication	We use a sample size of ten and then replicate the experiment three times to ensure the results are reproducible, when animals of the right genotypes, age/size are available.
Randomization	we take animals for the experiments randomly from clutches of 100 embryos, all animals look the same so ahve equal chance of beign selected for an experiment.
Blinding	Blinding is not necessary for these experiments, there was no behavorial tests involved. The results are clear results .

Reporting for specific materials, systems and methods

Materials & experimental systems

n/a	Involved in the study	
☒	☐	Unique biological materials
☐	☒	Antibodies
☐	☒	Eukaryotic cell lines
☒	☐	Palaeontology
☐	☒	Animals and other organisms
☒	☐	Human research participants

Methods

n/a	Involved in the study	
☒	☐	ChIP-seq
☒	☐	Flow cytometry
☒	☐	MRI-based neuroimaging

Antibodies

Antibodies used

beta-III tubulin antibody (mouse monoclonal)
Sigma
Clone: 2G10
Cat. No. T8578
Lot: N/A

c-Jun (Rabbit monoclonal)
Cell Signaling Technologies
Clone: 60A8
Cat. No.: 9165S
Lot: 9

GFAP (mouse monoclonal)
Millipore
Clone: GA5
Cat. No.: MAB360
Lot: 2898361

NeuN (mouse)
Millipore
Clone: N/A
Cat No:MAB377
Lot: 2654334

	c-Fos (Rabbit polyclonal)<br>Millipore<br>Clone: N/A<br>Cat. No.: ABE457<br>Lot: 2673548
Validation	c-Fos validation: validation from Millipore website:Evaluated by Western Blot in PMA(TPA) treated HeLa cell lysate.<br>NeuN validation from Millipore: Routinely evaluated by immunohistochemistry on brain tissue.<br><br>Immunohistochemistry(paraffin) Analysis:<br>NeuN (cat. # MAB377) staining pattern/morphology in rat cerebellum. Tissue pretreated with Citrate, pH 6.0. This lot of antibody was diluted to 1:100, using IHC-Select® Detection with HRP-DAB. Immunoreactivity is seen as nuclear staining in the neurons in the granular layer. Note that there is no signal detected in the nucleus of Purkinje cells.<br>Optimal Staining With Citrate Buffer, pH 6.0, Epitope Retrieval: Rat Cerebellum<br><br>GFAP: from Millipore website: Routinely evaluated by Western Blot on Mouse brain lysates.<br>Western Blot Analysis:<br>1:1000 dilution of this lot detected GFAP on 10 µg of Mouse brain lysates.<br><br>C-Jun: no validation information available on company website, validated in lab on cells overexpressing axolotl c-Jun.

Eukaryotic cell lines

Policy information about cell lines

Cell line source(s)	ATCC, cell sued are B35 (CRL-2754) and HEK cell (CRL 1573)
Authentication	cell lines were tested to express genes associated with those cell lines are indicated by the ATCC
Mycoplasma contamination	all cell lines tested negative for mycoplasma
Commonly misidentified lines<br>(See ICLAC register)	none of the cell lines used are listed as misidentified

Animals and other organisms

Policy information about studies involving animals; ARRIVE guidelines recommended for reporting animal research

Laboratory animals	Ambystoma mexicanum, white animals were used in all experiments. All experiments were done in accordance with IACUC protocols number No. 1710-35242A at UMN and IACUC protocol 18-49 at the Marine Biological Laboratory
Wild animals	N/A
Field-collected samples	N/A
